# Combined Effect of AMPK/PPAR Agonists and Exercise Training in *mdx* Mice Functional Performance

**DOI:** 10.1371/journal.pone.0045699

**Published:** 2012-09-21

**Authors:** Carlos R. Bueno Júnior, Lucas C. Pantaleão, Vanessa A. Voltarelli, Luiz Henrique M. Bozi, Patricia Chakur Brum, Mayana Zatz

**Affiliations:** 1 Human Genome Research Center - Institute of Biosciences, University of Sao Paulo, Sao Paulo, Brazil; 2 Department of Physiology and Biophysics - Institute of Biomedical Sciences, University of Sao Paulo, Sao Paulo, Brazil; 3 School of Physical Education and Sport, University of Sao Paulo, Sao Paulo, Brazil; Goethe University, Germany

## Abstract

The present investigation was undertaken to test whether exercise training (ET) associated with AMPK/PPAR agonists (EM) would improve skeletal muscle function in mdx mice. These drugs have the potential to improve oxidative metabolism. This is of particular interest because oxidative muscle fibers are less affected in the course of the disease than glycolitic counterparts. Therefore, a cohort of 34 male congenic C57Bl/10J mdx mice included in this study was randomly assigned into four groups: vehicle solution (V), EM [AICAR (AMPK agonist, 50 mg/Kg-1.day-1, ip) and GW 1516 (PPARδ agonist, 2.5 mg/Kg-1.day-1, gavage)], ET (voluntary running on activity wheel) and EM+ET. Functional performance (grip meter and rotarod), aerobic capacity (running test), muscle histopathology, serum creatine kinase (CK), levels of ubiquitined proteins, oxidative metabolism protein expression (AMPK, PPAR, myoglobin and SCD) and intracellular calcium handling (DHPR, SERCA and NCX) protein expression were analyzed. Treatments started when the animals were two months old and were maintained for one month. A significant functional improvement (p<0.05) was observed in animals submitted to the combination of ET and EM. CK levels were decreased and the expression of proteins related to oxidative metabolism was increased in this group. There were no differences among the groups in the intracellular calcium handling protein expression. To our knowledge, this is the first study that tested the association of ET with EM in an experimental model of muscular dystrophy. Our results suggest that the association of ET and EM should be further tested as a potential therapeutic approach in muscular dystrophies.

## Introduction

Duchenne muscular dystrophy (DMD) is an X-linked lethal genetic disease caused by the absence of the protein dystrophin [1]. This subsarcolemmal protein transmits the tension from the contractile proteins to the extracellular matrix and maintains the stability of the plasma membrane, avoiding the extrusion of intracellular constituents, including creatine kinase to blood serum, which is hallmark of the disease [2]. All patients have grossly increased serum CK levels [3]–[4] since birth, which decrease with the progression of the dystrophic process, reaching almost normal levels in the late stages. Affected boys are usually wheelchair-bound around age 10–12 and assisted ventilation is required in order to prolong survival after adolescence. The mdx mouse also lack muscle dystrophin and is the most widely used animal model for DMD [5].

Many investigators suggest that exercise training is beneficial in muscular dystrophies, since it results in better intracellular calcium handling, activation of compensatory or antagonistic signaling pathways, increased antioxidant capacity and angiogenesis, improved ability to blunt alpha-adrenergic vasoconstriction, increased iNOS activity and membrane protective effects [6]–[12]. However, others claim it has detrimental effects, which could be caused by the increased susceptibility of dystrophic muscle to exercise-induced injury. It is also known that these contradictory results are mainly related to type and intensity of exercise [13]–[15].

AMPK (AMP-activated protein kinase) and PPAR (peroxisome proliferator-activated receptor) agonists are exercise mimetics. In 2009, Miura et al. demonstrated that a PPAR agonist reduced the number of skeletal muscle fiber membrane lesions and decreased force drop due to eccentric contractions in mdx mice [16]. It has been demonstrated that these drugs are able to improve the oxidative metabolism - they induce mitochondrial biogenesis and fatty acid oxidation [17]. The improvement of the oxidative metabolism in muscular dystrophies is critical since it has been demonstrated, both in experimental models and humans, that slow oxidative muscles display reduced damage when compared to the glycolitic counterparts [18]–[19].

As exercise training and its mimetics present both coincident and different beneficial effects [17], the aim of the present study was to test the hypothesis that the association of exercise training with AMPK and PPAR agonists can improve mdx functional performance, reducing the impact of muscle dystrophin deficiency. In order to address this question, several parameters, such as functional tests, histopathology, skeletal muscle protein expression related to oxidative metabolism and calcium handling, renal function, and fat in the carcass, were analyzed in animals submitted or not to exercise.

**Table 1:** Basic parameters.

	V	EM	ET	EM+ET
	(9)	(8)	(9)	(8)
Body weight (g)	31.4±1.1	29.2±1.1	29.4±0.7	29.9±1.2
Food intake (g/day)	3.5±0.2	4±0.1	3.5±0.2	3.6±0.1
Feces (g/day)	2.4±0.1	2.7±0.2	2.1±0.1	2.2±0.1
Water intake (g/day)	3.2±0.4	2.9±0.3	3.1±0.2	2.9±0.2
Urine (g/day)	2±0.3	1.7±0.2	1.5±0.1	1.9±0.1
Glucose (mg/dl)	90.8±4.7	100.7±5.1	83.5±6.6	97±7.3
Triglycerides (mg/dl)	140±19	170±13	172±22	231±25*
Cholesterol (mg/dl)	110.3±5.6	104.5±4.4	117.8±7.7	115.9±4.8

The number of animals in each group is shown in parentheses and the data are presented as mean ± SE. *P<0.05 versus vehicle group (V) after two-way ANOVA and Duncan post-hoc test.

## Materials and Methods

### Ethics Statement

This study was conducted in accordance with the ethical principles in animal research adopted by the Brazilian College of Animal Experimentation (www.cobea.org.br) and was approved by the University of São Paulo, Institute of Biosciences Ethical Committee (087/2009).

### Study Population

A cohort of 34 two-months old male congenic C57Bl/10J mdx mice were randomly assigned into four groups: vehicle solution (V), exercise mimetics (EM), exercise training (ET) and EM+ET. Analysis started when the animals were three months-old. During all the month before the EM mice received AICAR (100 mg.Kg^−1^.day^−1^, ip, Cayman Chemical, catalog number 10010241, Ann Arbor, MI, USA) and GW 1516 (5 mg.Kg^−1^.day^−1^, gavage, Axxora, catalog number ALX-420-032-M005, San Diego, CA, USA) every other day and the ET animals stayed in individual cages with activity wheel and a cycle computer to register time and intensity of exercise. The mice were maintained in a 12:12 h dark-light cycle and temperature-controlled environment (22°C) with free access to tap water and standard laboratory chow (Nuvital Nutrientes S/A, Curitiba, PR Brazil). Before being killed, the animals stayed for 24 hours in metabolic cages for measurements of food intake, feces excretion, water intake and urine excretion. Furthermore, after a night fasting (one week before to be killed), their blood was harvested for glucose, triglycerides and cholesterol analysis (Labtest, Lagoa Santa, MG, Brazil).

**Figure 1:**
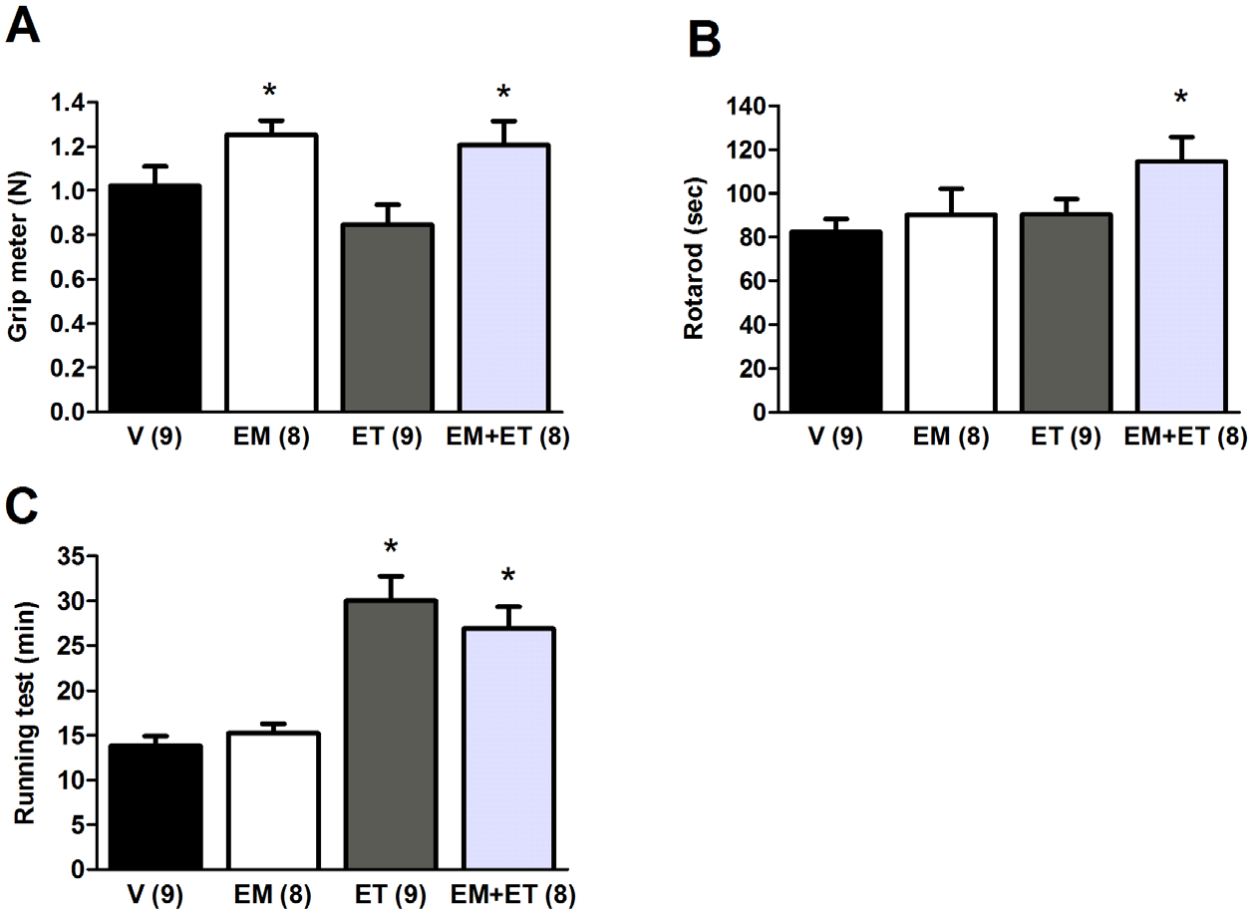
Exercise training associated with its mimetics causes the best improvements on functional capacity in mdx mice. Grip force (A), time until fall in the Rotarod evaluation (B) and time until exhaustion in the treadmill test (C) after one month of vehicle solution (V), exercise mimetics (EM), exercise training (ET) and EM+ET. The number of animals in each group is shown in parentheses and the data are presented as mean ± SE. *P<0.05 versus vehicle group (V) after two-way ANOVA and Duncan post-hoc test.

### Functional Tests

In order to evaluate the grip force, the animals were allowed to grab onto the Grip Strength System (model: DFE-002, San Diego Instruments, San Diego, Cal, USA) with the forepaws as the experimenter gently pulled on their tails - the result is the maximal force before the animal releases the forepaws of the bar (mean of three measurements of maximum pull) [20]. For the rotarod test, the equipment (model 755, IITC Life Science, Woodland Hills, CA, USA) was programmed to present an initial speed of 1 rpm and a final speed of 40 rpm, 300 seconds later. The result is the mean time that the animal is able to stay on the top of the gyratory rod - each animal performed three trials [21]–[23]. Finally, aerobic exercise tolerance was evaluated using a graded treadmill exercise protocol for mice, as previously described [24]. Briefly, after being accustomed to treadmill exercises over a week (10 min per day), mice were placed in the treadmill lane and allowed to acclimatize for at least 30 minutes. The intensity of exercise was increased by 3 m/min (6–33 m/min) every 3 min at 0% grade until exhaustion.

### Muscle Histopathology

After the tests, the mice were killed and triceps braquialis muscle was harvested, immediately frozen in melting isopentane and stored in liquid nitrogen. The frozen muscles were cut into 10-µm cross sections from the proximal to distal region using a cryostat (Criostat Mícron HM505E, Walldorf, Germany). Sections of muscle were then stained with hematoxylin and eosin [25] or used to perform histochemical myosin ATPase [26], as previously described. The cross-sectional area of the fibers was evaluated at 200x magnification and further analyzed on a digitizing unit connected to a computer (Image Pro-plus, Media Cybernetic, Silver Spring, MD, USA). The cross-sectional area and the percentage of interstitium and type I fibers in the muscle were analyzed in blind test. In addition, serum creatine kinase was analyzed when the animals were three months old, when normal values for control animals are reported to be low (around 100 U/l) [27].

### Skeletal Muscle Protein Expression

In order to evaluate aspects related to muscular structure, oxidative metabolism and calcium handling, immunoblots of triceps braquialis muscle homogenates were performed according to Bacurau et al. [26]. Briefly, frozen muscles in liquid nitrogen were homogenized in a buffer containing 1 mM EDTA, 1 mM EGTA, 2 mM MgCl2, 5 mM KCl, 25 mM HEPES (pH. 7.5), 100 µM PMSF, 2mM DTT, 1% Triton X-100 and protease inhibitor cocktail (1∶100, Sigma-Aldrich, MO, USA). Samples were loaded and subjected to SDS-PAGE in polyacrylamide gels. After electrophoresis, proteins were electro-transferred to nitrocellulose membrane (Amersham Biosciences, NJ, USA). Equal loading of samples (25 µg) and even transfer efficiency were monitored using 0.5% Ponceau S staining of the blotted membrane. The blotted membrane was then incubated in a blocking buffer (5% nonfat dry milk, 10 mM Tris-HCl, pH 7.6, 150 mM NaCl, and 0.1% Tween 20) for 2 h at room temperature and then incubated overnight at 4°C with the following primary antibodies: ubiquitined proteins (1∶1000) from Santa Cruz Biotechnology; AMPKα (1∶1000), p-AMPKα (Thr172, 1∶1000), ACC (1∶1000) and p-ACC (Ser79, 1∶1000) from Cell Signaling Technology; PPARδ (1∶1000), Myoglobin (1∶1000) and SCD (1∶1000) from Abcam; and DHPR-α1 (1∶1000), SERCA 1 (1∶1000) and NCX (1∶1000) from ABR Incorporation. Binding of the primary antibody was detected using peroxidase-conjugated secondary antibodies (anti-mouse or anti-rabbit, 1∶3000, for 90 minutes at room temperature) and developed using enhanced chemiluminescence (Amersham Biosciences; Piscataway, NJ, USA) detected by autoradiography. Quantification analysis of blots was performed using Scion Image software (Scion Corporation based on NIH image).

**Figure 2:**
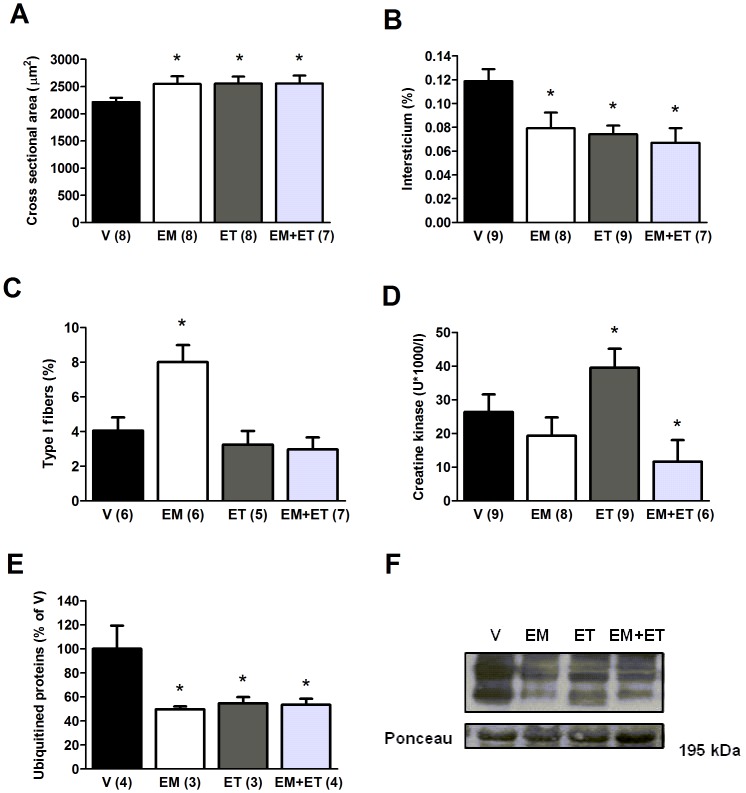
When associated with its mimetics, exercise training does not impair skeletal muscle structure in mdx mice. Cross sectional area (A), intersticium (B), percentage of type I muscle fibers (C), blood creatine kinase (D) and ubiquitined proteins (E) after one month of vehicle solution (V), exercise mimetics (EM), exercise training (ET) and EM+ET. The number of animals in each group is shown in parentheses and the data are presented as mean ± SE. *P<0.05 versus vehicle group (V) after two-way ANOVA and Duncan post-hoc test.

### Creatinine Clearance and Fat in the Carcass

In order to evaluate mice renal function, we evaluated creatinine clearance [28]. Carcass fat was determined by its chemical analysis. Initially, carcasses stayed in a ventilated oven (70°C) for 7 days. Then, whole dry carcass was chopped up and wrapped in gauze and filter paper for determination of body fat by the solvent extraction technique using a Soxhlet apparatus and ethyl ether as solvent [29].

### Statistical Analysis

All values are presented as means ± SE. Two-way analysis of variance (ANOVA; exercise mimetics and exercise training as variables) and Duncan post-hoc test (Statistica software, StatSoft, Inc., Tulsa, OK, USA) were used to compare the groups. Statistical significance was considered as p≤0.05.

**Figure 3:**
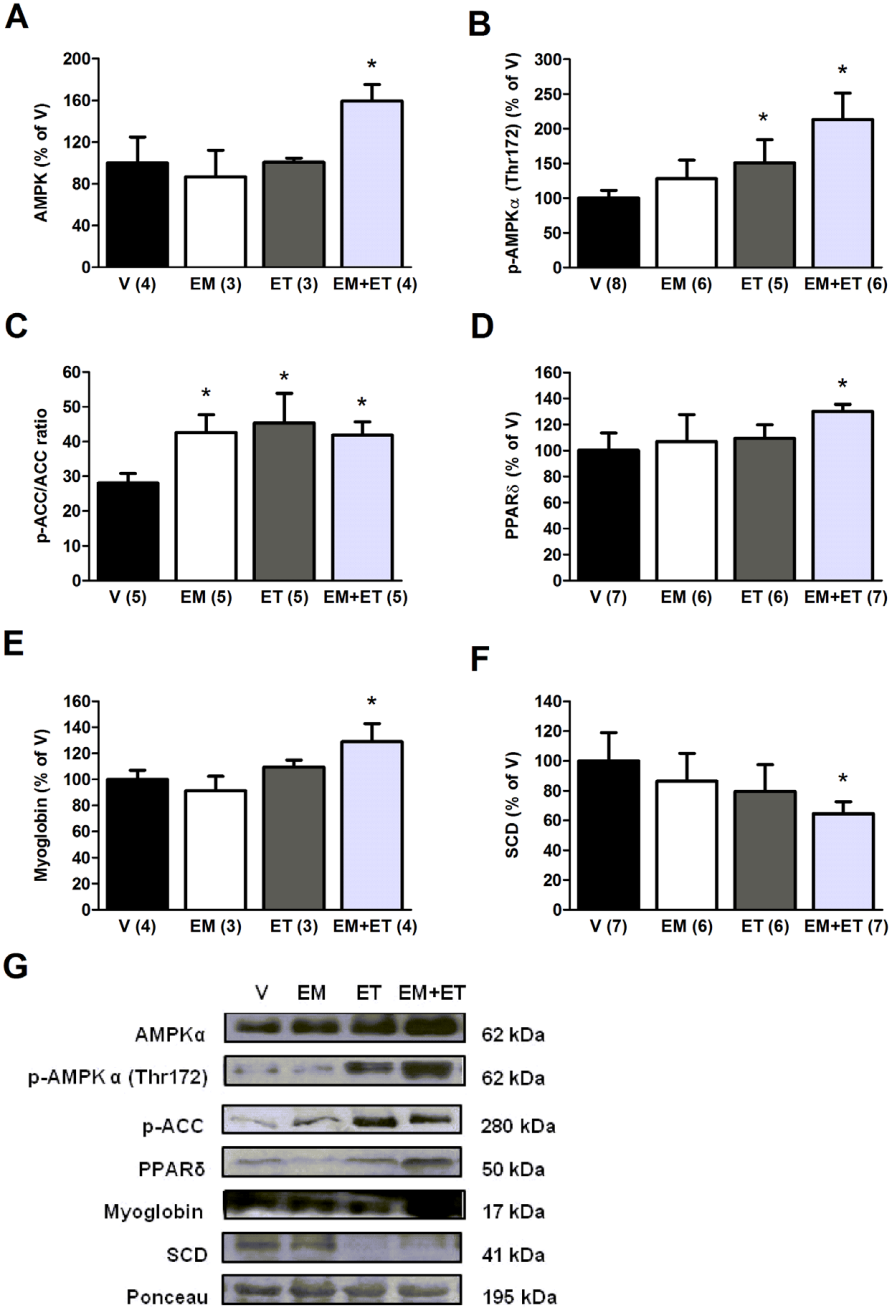
Exercise training associated with its mimetics improves protein expression related to the oxidative metabolism. Muscular protein expression of AMPKα (A), p-AMPKα(Thr172) (B), p-ACC/ACC ratio (C), PPARδ (D), Myoglobin (E) and SCD (F) after one month of vehicle solution (V), exercise mimetics (EM), exercise training (ET) and EM+ET. The number of animals in each group is shown in parentheses and the data are presented as mean ± SE. *P<0.05 versus vehicle group (V) after two-way ANOVA and Duncan post-hoc test.

## Results

### Basic Parameters

As presented in the Table 1, there was no statistical difference among the groups in body weight, food intake, feces excretion, water intake and urine excretion. In addition, the data of serum glucose and cholesterol after night fasting were similar in the groups. Only triglycerides levels were increased in the group that received exercise mimetics and exercise training when compared to the values of the vehicle group.

**Figure 4:**
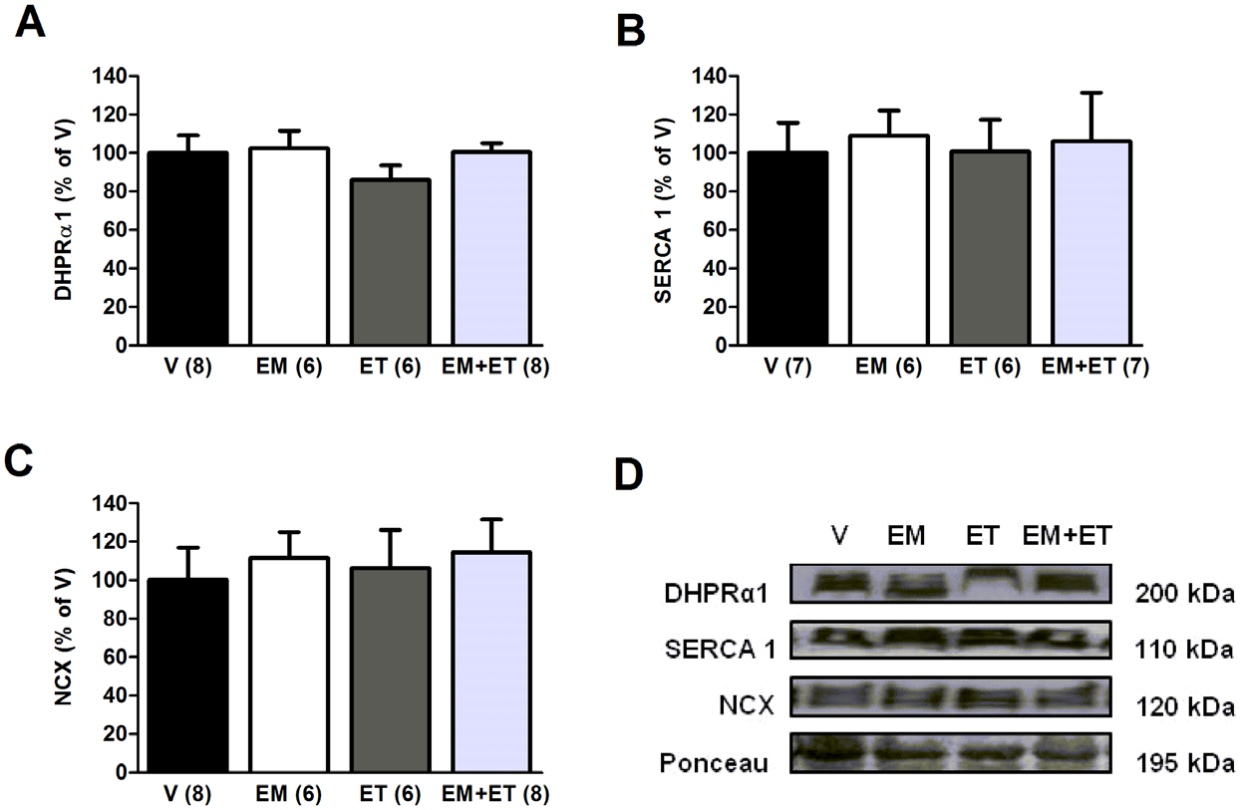
Exercise training and its mimetics do not change calcium handling protein expression. DHPRα1 (A), SERCA 1 (B) and NCX (C) protein expression after one month of vehicle solution (V), exercise mimetics (EM), exercise training (ET) and EM+ET. The number of animals in each group is shown in parentheses and the data are presented as mean ± SE. *P<0.05 versus vehicle group (V) after two-way ANOVA and Duncan post-hoc test.

### Functional Capacity

Figure 1 shows that the group submitted to both exercise mimetics and exercise training presented improved functional performance (grip meter - Figure 1A, p = 0.05; and rotarod - Figure 1B, p = 0.009) as well as aerobic capacity (running test - Figure 1C, p<0.001), which was statistically significant when compared to the vehicle group. Exercise mimetics only ameliorated significantly grip force (Figure 1A, p = 0.026) and exercise training only improved significantly aerobic capacity (Figure 1C, p<0.001). Of interest, during the entire month of treatment activity wheel was moved 2.9±0.4 hours per day in a speed of 9±0.6 m/min by the exercise training group. These mean values were 2.8±0.5 hours and 5.8±0.6 m/min, respectively, for the group that received both interventions (there is a statistical difference between 9±0.6 m/min and 5.8±0.6 m/min, p = 0.004).

**Figure 5:**
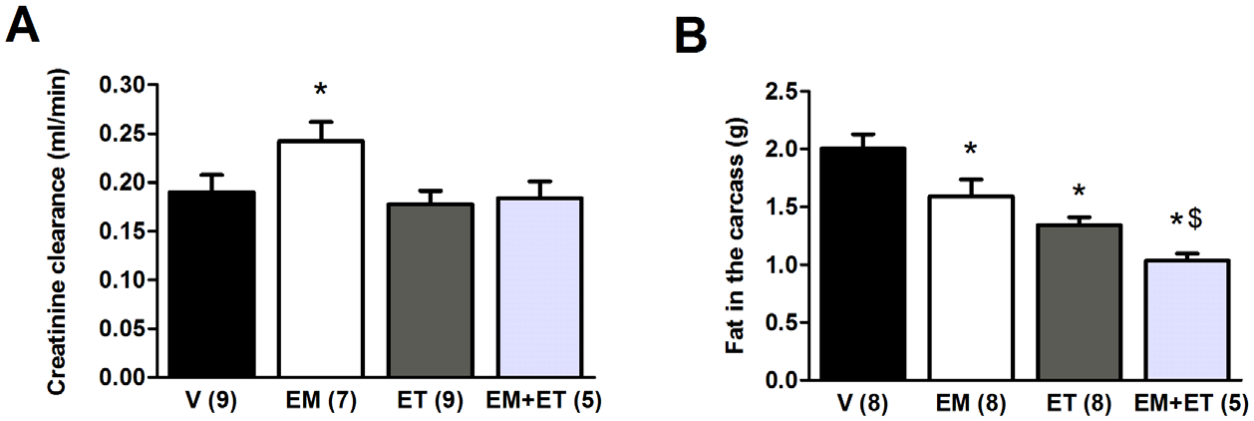
Exercise training associated with its mimetics does not impair renal function and reduces fat in the carcass. Creatinine clearance (A) and fat in the carcass (B) after one month of vehicle solution (V), exercise mimetics (EM), exercise training (ET) and EM+ET. The number of animals in each group is shown in parentheses and the data are presented as mean ± SE. *P<0.05 versus vehicle group (V) after two-way ANOVA and Duncan post-hoc test. ^$^P<0.05 versus EM and ET groups.

### Skeletal Muscle Histology, Serum Creatine Kinase and Ubiquitined Proteins

In animals that had a better functional capacity (exercise mimetics + training), skeletal muscle showed statistically increased cross sectional area (Figure 2A, p = 0.024) associated with reduced interstitium (Figure 2B, p = 0.003), serum creatine kinase (Figure 2C, p = 0.048) and ubiquitined proteins (Figure 2D, p = 0.029) as compared to the vehicle group. Exercise training alone led to significantly augmented fibers (Figure 2A, p = 0.023) and reduced percentage of interstitium and ubiquitined proteins levels (Figure 2B and D; p = 0.001 and 0.05, respectively), but increased levels of blood creatine kinase (Figure 2C, p = 0.05). Exercise mimetics alone, on the other hand, statistically improved cross sectional area (Figure 2A, p = 0.028), percentage of interstitium (Figure 2B, 0.015), percentage of type I fibers (Figure 2C, p = 0.003) and levels of ubiquitined proteins (Figure 2D, p = 0.04), with no change on creatine kinase levels (Figure 2C).

### Protein Expression Related to Oxidative Metabolism and Calcium Handling

Since an improvement in oxidative metabolism is a proposed mechanism to explain the beneficial effects of both exercise mimetics and exercise training, we evaluated the expression of proteins related to oxidative metabolism. As observed in Figure 3, the association of both approaches increased the expression of key proteins (p = 0.046 for AMPKα, p = 0.003 for p-AMPKα, p = 0.009 for p-ACC/ACC ratio, p = 0.031 for PPARδ and p = 0.05 for myoglobin) and the activity of one of them (AMPKα, p = 0.003), assessed by phosphorylation levels (Figure 3A–E). Interestingly, in this group the expression of SCD, a pivotal enzyme in lipogenesis, was decreased (Figure 3F, p = 0.05) when compared to the control group. However, when we analyzed exercise training and its mimetics isolatedly, there was a significant increase only in the level of AMPKα phosphorylation in the exercise training group (Figure 3B, p = 0.05) and in the p-ACC/ACC ratio in both groups (Figure 3C, p = 0.018 for exercise mimetics group and p = 0.044 for wheel activity group).

Since impaired transsarcolemmal calcium flux and sarcoplasmic reticulum calcium release/reuptake has been identified as main contributors to skeletal muscle functional and structural abnormalities [30], we further investigated whether exercise mimetics and exercise training would change the expression of DHPR, SERCA and NCX. However, as shown in Figure 4, there was no statistical difference among the groups studied.

### Renal Function and Fat in the Carcass

As a potential adverse effect of different drugs can be an impaired renal function, we analyzed creatinine clearance. Interestingly, the exercise mimetics group displayed better renal function than the other groups (Figure 5A, p = 0.033). There was no difference among the other groups (Figure 5A).

Finally, as both aerobic exercise training and AMPK/PPAR agonists accelerate the oxidative metabolism, an expected consequence would be a reduction in the amount of fat in the body. In order to test this hypothesis, we analyzed the carcass fat of the animals. The results are presented in the Figure 5B. When compared to the vehicle group, all three approaches reduced the amount of fat in mice (p = 0.024 for the exercise mimetics group and p<0.001 for the exercise training and combined therapy groups), but the group that received both exercise mimetics and exercise training showed a statistically significant decrease when compared to the groups submitted to the isolated approaches (p = 0.007 for the exercise mimetics group and p = 0.005 for the exercise training group).

## Discussion

The main result of the present study was that voluntary exercise training combined with AMPK/PPAR agonists improves the functional performance and the aerobic capacity of mdx mice. Of particular interest is the fact that these improvements might be associated to reduced muscle degeneration (increased muscle fiber cross sectional area and diminished ubiquitined proteins expression) and improved efficiency of the aerobic metabolism. To our knowledge, this is the first study that tested the association of exercise training with AMPK/PPAR agonists in a disease murine model.

Here we show that the association of exercise training and AMPK/PPAR agonists resulted in functional improvements related to grip meter, rotarod and aerobic capacity (running test). The animals submitted only to the exercise training showed improved aerobic capacity, supporting the efficiency of the training, and the animals that received only the drugs had an improvement in the grip meter test. Our data in the running test corroborated Narkar et al. [17], who reported that the association of AICAR and exercise training improved the running capacity in control mice.

Interestingly, the exercise training group presented increased values of training intensity in wheel activity when compared to the animals that also received exercise mimetics - the time of activity per day was similar in both groups. This can help explain, at least in part, the results where the combined therapy did not result in a better outcome as compared to the isolated approaches. Although different intensities between the groups can be a limitation of the present study, we did the running test before the treatment to certify that both groups presented similar aerobic capacity (data not shown). Furthermore, the absence of exercise intensity control is inherent to the activity wheel method and this kind of voluntary training was chosen because it has been demonstrated that it avoid excessive overload to the dystrophic muscle. Exercise intensity is a prominent factor to induce muscle adaptations, but dystrophic patients are not able to perform intense exercises because their muscles are more susceptible to mechanical stress [31].

Although strength training is more efficient to induce hypertrophic response, aerobic exercise training also can increase muscle mass, particularly in animals with skeletal muscle abnormalities [26], [32]. In addition, related to the exercise mimetics group, it is possible that AMPK/PPAR agonists might increase protein synthesis through higher metabolism efficiency and reducing the need of proteolysis to generate energy by gluconeogenesis. In fact, both groups that received AMPK/PPAR agonists had decreased ubiquitined proteins - the ubiquitination of proteins has a direct relationship with the activity of the ubiquitin-proteasome system, the main mechanism of protein breakdown in the cell [33].

As stated before, glycolitic muscle fibers are more affected by the dystrophy when compared to the oxidative counterparts and only the exercise mimetics group presented increased percentage of type I fibers than the control group. This effect of drugs is in accordance with Narkar et al. [17]. Furthermore, the observation that exercise training is unable to increase the percentage of oxidative fibers corroborated other studies [34]. One possible explanation is that the intensity of the exercise was excessive for the dystrophic mice, which would also explain the higher levels of serum CK in the exercise training group. It was possible to observe that the animals performed high intensity exercises to move the wheel, which continued in movement by inertia. Although one month can be insufficient to result in changes in the type fiber profile, serum CK may increase rapidly after exercise. Finally, in the animals that received both treatments the high intensity exercise could have caused a negative interference in the effect of the exercise mimetics, which resemble aerobic exercise [35].

We have no explanation for the apparently decreased activity in serum CK in the mimetics group. It could be due to the small sample size or to the natural fluctuation that is seen in this enzyme which might not be directly related to the dystrophic process [36].

We also observed that in the group in which exercise training was associated with AMPK/PPAR agonists, there was an increased expression of AMPK, phosphorylated AMPK and PPARδ. The exercise training group also presented increased AMPK activity evaluated by phosphorylation and these results corroborate the running test data since both groups with highest running performance presented increased AMPK activity. Additionally, it is known that mice with dysfunction in the skeletal muscle AMPK signaling present reduced running capacity [37]–[38] and many studies have demonstrated that exercise training increases the AMPK activation [17], [28], [39]. Finally, the group submitted to both exercise training and its mimetics displayed increased myoglobin protein expression - a protein that has pivotal importance in the storage of oxygen in the muscle cell, especially in dystrophic muscle fiber, in which myoglobin can leak from the cell cytosol due the disruption of plasma membrane [40]. This group also presented reduced levels of SCD, a key enzyme in the lipogenesis [41]. In fact, in this set of animals the levels of triglycerides were increased.

However, the high levels of triglycerides in this group do not represent a metabolic disorder - these animals do not present increased cholesterol and glucose values, for example. The result can be related to decreased lipogenesis (discussed before) and the time that blood was harvested for analysis. In addition, Narkar et al. demonstrated that triglycerides levels may be increased by exercise [17].

Although they were not analyzed in previous studies with AMPK/PPAR agonists [16]–[17], potential adverse effects related to exercise mimetics should be monitored carefully. Interestingly, the exercise mimetics group showed improved renal function by creatinine clearance test. Finally, we showed that all strategies reduced carcass fat, but with higher magnitude in the animals submitted to both exercise training and AMPK/PPAR agonists. This result suggests increased lipolysis and generation of energy by the oxidative metabolism, which is supported by the increased serum levels of triglycerides in this group. The significance of the reduction in the carcass fat is highlighted if we consider that there was no statistical difference among the groups in body weight.

We also demonstrated that the ratio between protein expressions of phosphorylated and total acetyl-CoA carboxylase (Ser79) was increased in the three groups submitted to the treatments, which indicate inhibition of carboxylation of acetyl-CoA to malonyl-CoA in the biosynthesis of fatty acids. These results might explain the results from the carcass fat. In addition, these data reinforce the effectiveness of the exercise mimetics treatment because an AMPK downstream activity is the phosphorylation of acetyl-CoA carboxylase [42].

In short, a beneficial effect of combination of exercise training and AMPK/PPAR agonists was observed in mdx mice. Although exercise training alone or alternatively its mimetics showed some improvement in skeletal muscle histology and aerobic capacity of mdx mice, the combination of both strategies seems more effective. In addition, our results suggest that a favorable protein turnover (represented by decreased levels of ubiquitined proteins and increased cross sectional area) as well an improved efficiency of the oxidative metabolism might explain, at least in part, the observed functional improvements. It will be interesting to repeat these studies in other experimental models of muscular dystrophy. Although most dystrophic patients are not able to run, the animals in the present study are in the early stages of dystrophy, when many children would still be able to exercise. It would also be of interest to assess if the association of passive exercise and AMPK/PPAR agonists have a comparable beneficial effect.
